# A new model construction based on the knowledge graph for mining elite polyphenotype genes in crops

**DOI:** 10.3389/fpls.2024.1361716

**Published:** 2024-03-20

**Authors:** Dandan Zhang, Ruixue Zhao, Guojian Xian, Yuantao Kou, Weilu Ma

**Affiliations:** ^1^ Agricultural Information Institute of Chinese Academy of Agricultural Sciences, Beijing, China; ^2^ Key Laboratory of Agricultural Integration Publishing Knowledge Mining and Knowledge Service, National Press and Publication Administration, Beijing, China; ^3^ Key Laboratory of Agricultural Big Data, Ministry of Agriculture and Rural Affairs, Beijing, China

**Keywords:** polyphenotype gene, cross-species, traits regulating-genes, knowledge graph, crop

## Abstract

Identifying polyphenotype genes that simultaneously regulate important agronomic traits (e.g., plant height, yield, and disease resistance) is critical for developing novel high-quality crop varieties. Predicting the associations between genes and traits requires the organization and analysis of multi-dimensional scientific data. The existing methods for establishing the relationships between genomic data and phenotypic data can only elucidate the associations between genes and individual traits. However, there are relatively few methods for detecting elite polyphenotype genes. In this study, a knowledge graph for traits regulating-genes was constructed by collecting data from the PubMed database and eight other databases related to the staple food crops rice, maize, and wheat as well as the model plant *Arabidopsis thaliana*. On the basis of the knowledge graph, a model for predicting traits regulating-genes was constructed by combining the data attributes of the gene nodes and the topological relationship attributes of the gene nodes. Additionally, a scoring method for predicting the genes regulating specific traits was developed to screen for elite polyphenotype genes. A total of 125,591 nodes and 547,224 semantic relationships were included in the knowledge graph. The accuracy of the knowledge graph-based model for predicting traits regulating-genes was 0.89, the precision rate was 0.91, the recall rate was 0.96, and the F1 value was 0.94. Moreover, 4,447 polyphenotype genes for 31 trait combinations were identified, among which the rice polyphenotype gene *IPA1* and the *A. thaliana* polyphenotype gene *CUC2* were verified via a literature search. Furthermore, the wheat gene *TraesCS5A02G275900* was revealed as a potential polyphenotype gene that will need to be further characterized. Meanwhile, the result of venn diagram analysis between the polyphenotype gene datasets (consists of genes that are predicted by our model) and the transcriptome gene datasets (consists of genes that were differential expression in response to disease, drought or salt) showed approximately 70% and 54% polyphenotype genes were identified in the transcriptome datasets of Arabidopsis and rice, respectively. The application of the model driven by knowledge graph for predicting traits regulating-genes represents a novel method for detecting elite polyphenotype genes.

## 1 Introduction

The development of the seed industry, which is critical for ensuring food security and maintaining the supply of important agricultural products, is largely dependent on high-quality crop gene resources. In terms of crop breeding, there is often a trade-off between traits. For example, there are negative correlations between high yield and disease resistance as well as between high yield and high quality. Thus, it may be difficult to simultaneously optimize two elite traits, which is one of the bottlenecks in crop breeding programs. Thus, mining for polyphenotype genes associated with elite traits may eliminate the trade-off effect during the development of new high-quality crop varieties. The “Breeding 4.0” concept is highlight the integration of life science, information science, and breeding science, which helps the identification of elite polyphenotype genes on the basis of hypothesis-driven passive exploration transition to data-driven active knowledge discovery (Wallace et al., 2018). In addition, the methods of genome-wide association study (Liu et al., 2023b), quantitative trait locus analysis (Jiang et al., 2023), and bulk segregant analysis (Yu et al., 2023) were conducted to establish the associations between genes and individual traits, but they were not conducive to mining for polyphenotype genes (Polderman et al., 2015; Sonah et al., 2015).

The rapid development and application of high-throughput sequencing technology has resulted in an exponential increase in the amount of multi-dimensional scientific data relevant to breeding. Moreover, several data platforms have emerged to support crop breeding research, including the genome annotation data platform Phytozome (Goodstein et al., 2012), the protein sequence and functional analysis platform UniProt (Unified Protein Database) (Consortium, 2007), the pathway annotation data platform KEGG (Kyoto Encyclopedia of Genes and Genomes) (Chen et al., 2017), the rice genomic variation and functional annotation platform RiceVarMap (Rice Variation Map) (Zhao et al., 2021), the wheat genome platform IWGSC (International Wheat Genome Sequencing Consortium) (Appels et al., 2018), and MaizeGDB (Maize Genetics and Genomics Database) (Portwood et al., 2019). These data platforms are useful for analyzing the molecular mechanism regulating traits, but typically only in a single dimension. However, the characteristics of multi-source heterogeneous data make it extremely difficult to integrate multi-dimensional scientific data, which is problematic when mining for elite polyphenotype genes. Hence, a new method for integrating multi-dimensional scientific data for genes and related traits is urgently needed to facilitate the mining and application of elite polyphenotype genes.

Knowledge graphs are constructed to organize knowledge using graphical representations for associated data and integrate diverse scientific data from multiple sources. The intrinsic relevance of the knowledge can be inferred by predicting relationships between entities in the knowledge graph (Himmelstein et al., 2017). Therefore, knowledge graphs have the ability to correlate and integrate multi-dimensional scientific data, thus enabling more effective exploration of subject knowledge discovery (Lan et al., 2021; Yang et al., 2022). Consideration of the data complex in life sciences, recently, a larger number of studies have focused on the application of knowledge graphs in medical sciences. Firstly, knowledge graphs applied in analysis of disease genes. Alshahrani et al. developed Semantic Disease Gene Embeddings (SmuDGE) to rank disease-related genes, this model was constructed based on the knowledge graph with using feature learning-embedding entity vectors (Alshahrani and Hoehndorf, 2018). In addition, a disease knowledge graph CADA (i.e., case annotations and disorder annotations) was constructed by Peng et al., which prioritizes the pathogenicity-related genes by combing the methods of network representation learning and link predictions (Peng et al., 2021). To mine the highly correlated cancer genes, Choi et al. proposed a knowledge graph-embedding model (KGED) based on convolutional neural networks, which used the gene-gene relationships inferred by KGED to generate gene interaction networks for specific cancer types (Choi and Lee, 2021). Secondly, knowledge graphs also applied in the discovery of knowledge paths in medical sciences. For instance, Dharmavaram et al. revealed the hidden knowledge discovery by using a random walk algorithm, which extracts contextual information from the knowledge graph and generates knowledge paths closely related to the input knowledge (Dharmavaram et al., 2019). Additionally, Pyysalo et al. delved the knowledge discovery in pathogenic mechanisms underlying tumor formation by combing the methods of machine learning and natural language processing, to analyze the strength of entity associations according to the knowledge discovery method involving the co-occurrence of concepts (Pyysalo et al., 2019). Thirdly, knowledge graphs were applied to the proposition of new research directions in medical sciences. Mohamed et al. constructed a knowledge graph-embedding semantic model for predicting new drug-target interactions, which exploited the existing drug-target knowledge graph DrugBank (Mohamed et al., 2020). In addition, Zhang et al. proposed a knowledge graph to support the discovery of new Parkinson’s drug candidates, which integrates knowledge from a local medical knowledge database and medical literature (Zhang and Che, 2021). Furthermore, Yang et al. constructed a stroke-related knowledge graph by applying biomedical text mining methods, this model was target to the prediction of new directions in stroke research or the reusing of related drugs (Yang et al., 2022). However, few of studies were applied the knowledge graphs in agricultural sciences. The Rothamsted Research Institute constructed the domain knowledge graph which is named KnetMiner, this model is focus on the knowledge system query of gene regulatory networks (Hassani-Pak et al., 2021). In addition, The French Agricultural Research Centre for International Development (CIRAD) constructed the knowledge graph of Agronomic Linked Data (AgroLD), which integrates multiple plant datasets to support the development of scientific hypotheses related to complex plant traits (Larmande et al., 2022). Hence, how to develop gene mining model based on knowledge graph, especially for mining elite polyphenotype genes remains largely unknown.

It is currently difficult to identify and determine the genomic locations of unknown genes using existing bioinformatics methods. Additionally, candidate genes must be individually verified via molecular biology experiments, which is often an expensive and time-consuming process. Furthermore, the proven methods for genome-wide association analyses, quantitative trait locus mapping, and mixed pool group analyses can establish the relationships between genes and single traits, but there are not appropriate for identifying elite polyphenotype genes. Thus, in this study, we constructed a model for predicting traits regulating-genes using a knowledge graph that combined the data attribute information of the gene nodes and the related topological relationship attribute information. Our objective was to predict traits regulating-genes on the basis of a multi-dimensional scientific data analysis, ultimately leading to the identification of elite polyphenotype genes.

## 2 Data and methods

### 2.1 Data sources

In our previous study, PubMed database was used as the literature data source, and 8 other domain knowledge bases were selected as data sources, including Phytozome, Ensemble (European molecular biology laboratory’s European bioinformatics institute), RGAP (rice genome annotation project), UniProt (universal protein resource), STRING (search tool for recurring instances of neighboring genes), Pfam (protein family), KEGG (Kyoto encyclopedia of genes and genomes) and GO (gene ontology) (Zhang et al., 2024).

All the transcriptome datasets were obtained from the PubMed database according the phenotypes. To obtain transcriptome datasets related to disease, we obtained 589 differentially expressed genes (DEGs) resistance to Verticillium dahlia in *Arabidopsis* (Scholz et al., 2018) and 4471 DEGs resistance to P kururiensisin in rice (King et al., 2019). In addition, we obtained 14165 and 9751 DEGs in response to salt in *Arabidopsis* and rice, respectively (Zhou et al., 2016; Crawford et al., 2020). And we also obtained 3341 and 3003 DEGs in response to drought in *Arabidopsis* and rice, respectively (Fu et al., 2017; Sura et al., 2017). All those DEGs (|log(Fold change)|≥1) were selected by comparison of the stress treatment groups and control groups in the wild type.

### 2.2 Construction of a knowledge graph for traits regulating-genes

Knowledge graph is a graph-based data structure consisting of multiple triples (entity-object attribute-entity) with nodes and edges, each node representing an “entity”, each edge representing an “object attributes”. Entity object attributes can reveal the semantic relationship between two entities.

In our previous studies, the knowledge graph for traits regulating-genes describes the relational hierarchy of knowledge organization between genes and traits. Its construction steps include the construction of ontology semantic model and triplet extraction based on ontology semantic model. Firstly, taking trait, gene and protein as the central entity, 13 entities are organized and associated through 14 object attributes to form an ontology semantic model. Secondly, based on the ontology semantic model, the corresponding entities are extracted from the above multiple data source knowledge base to form multiple triples. Finally, the constructed multi-type triplets was stored in the Neo4j graph database to form the knowledge graph for traits regulating-genes (Zhang et al., 2024).

### 2.3 Construction of a model for predicting traits regulating-genes

In knowledge graph, knowledge mining method of link prediction is that the likelihood of a “new link” between entities may be predicted by calculating the closeness between two entities. The study have shown that link prediction can be applied in knowledge graph to realize the subject knowledge discovery (Peng et al., 2021). Based on the knowledge mining principle of link prediction, we constructed a model for predicting traits regulating-genes.

Considering the unknown relationships between genes and traits, this study adopted the knowledge mining method based on predicted links to determine the similarity between the entities of unknown genes (unknown relationships between the genes and traits) and known genes (known relationships between the genes and traits). Potential “new links” between unknown genes and traits were predicted according to the traits regulated by known genes. The characteristics of the multi-dimensional scientific data associations between the genes and traits in the knowledge graph were used to construct a model for predicting traits regulating-genes. The model calculates the similarity between known gene and unknown gene entities to predict the traits regulated by the unknown genes.

**Figure 1:**
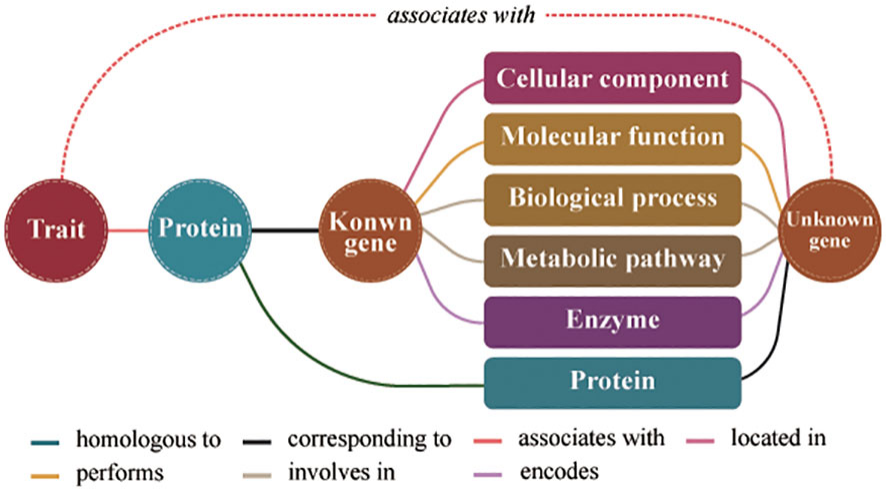
Schematic diagram of the model for predicting traits regulating-genes. Different colored lines represent different relationships between entities.


The formula for assessing the similarity between two gene entities in the model consists of three parts (**Figure 1**). First, the number of co-connected entities between genes represents the co-occurrence frequency of unknown genes and known genes. Second, the number of co-connected entity categories between genes represents the structural characteristics shared by unknown genes and known genes at different molecular levels. Third, the similarity score between the proteins obtained by calculating protein sequence similarity. The similarity score represents the genes similarity in conserved functions. Therefore, we predicted the traits regulating-genes using the following formula for calculating the similarity score:

S(g1, g2)=C(k)·D(k)·S(p1, p2).

where S(g1, g2) indicates the degree of similarity between two gene entities; g1 is a known trait regulating-gene; g2 is an unknown trait regulating- gene; S(p1, p2) indicates the degree of similarity between two protein entities; p1 and p2 respectively are the proteins encoded by g1 and g2; N(x) represents a collection of nodes adjacent to the node gene x; *k*=N(g1)∩N(g2); C(*k*) is the number of entities in the node collection *k*; and D(*k*) is the number of entity categories for the node collection *k*. In the formula, C(k) and D(k) represent topological relationship attributes of the gene nodes in the knowledge graph, whereas S(p1, p2) represents data attribute of the gene nodes in the knowledge graph.

### 2.4 Determination of the threshold for the model for predicting traits regulating-genes

The model for predicting traits regulating-genes was used to quantify the similarity between unknown genes and known genes. A high similarity score indicates that the unknown gene is closely related to a known gene. Moreover, the genes may regulate the same trait. We assumed that when the similarity score exceeded a certain threshold, the unknown gene and the known gene regulate the same trait. To determine the threshold, the gene-trait association datasets published from 1988 to 2023 were selected to make the gene-gene association datasets, which to calculate the similarity to all known trait regulating- genes. Based on the time when genes were first discovered, gene-gene association datasets were divided into training sets and validation sets in a ratio about 8:2. The gene-gene association datasets compiled from 1988 to 2017 of a total of 728 served as the training set, whereas the gene-gene association datasets compiled from 2018 to 2023 of a total of 157 served as the validation set. The threshold was determined by training the datasets, with the F1 value used as the threshold filtering index. More specifically, the similarity score corresponding to the maximum F1 value was set as the threshold (Powers, 2020).

### 2.5 Evaluation of the utility of the model for predicting traits regulating-genes

On the basis of the selected threshold, the time-slicing method was used to predict the regulatory genes for traits in the test set. Four commonly used evaluation indices (i.e., accuracy, precision, recall, and F1 value) were used to evaluate the screening results in the validation set to verify the utility of the model. The formulae for the selected indices were as follows:


$$\text{Accuracy}=\frac{\text{TP}+\text{TN}}{\text{TP}+\text{TN}+\text{FP}+\text{FN}},$$

$$\text{Precision}=\frac{\text{TP}}{\text{TP}+\text{FP}},$$

$$\text{Recall}=\frac{\text{TP}}{\text{TP}+\text{FN}}\;\text{and}$$

$$\text{F}1-\text{score}=\frac{2\times \text{Precision}\times \text{Recall}}{\text{Precision}+\text{Recall}},$$

where True Positive (TP) is an outcome where the model correctly predicts the positive class; True Negative (TN) is an outcome where the model correctly predicts the negative class; False Positive (FP) is an outcome where the model incorrectly predicts the positive class; False Negative (FN) is an outcome where the model incorrectly predicts the negative class.

### 2.6 Mining for polyphenotype genes affecting the target traits

The model for predicting traits regulating-genes was used to examine the similarity between the unknown genes and the known genes regulating the target traits. The known genes that satisfied the threshold were selected to predict the traits regulated by the unknown genes. Finally, the associations between the unknown genes and multiple traits were established to identify the polyphenotype genes regulating the target traits. Increases in the similarity score reflected increases in the likelihood the unknown gene and known gene regulate the same trait. Considering the differences in the distribution of the similarity scores, we propose a comprehensive ranking method of elite polyphenotype genes. We comprehensively ranked the elite polyphenotype genes by ranking the unknown gene similarity scores for each regulated trait and then ranking the polyphenotype genes by counting their average ranking in each regulated trait. The following formula was used for this ranking:


$$S_{sum}=\sum_{i=1}^{n}\frac{R\left(g_{i}\right)}{n}.$$

where *i* represents the traits; *n* represents the number of traits; and *R*(*g_i_*) represents the similarity score ranking for the gene for the *i* trait.

## 3 Results

### 3.1 Knowledge graph for traits regulating-genes

Data for the model plant *Arabidopsis thaliana* and the staple crops rice, maize, and wheat were used to construct a knowledge graph comprising 13 entities, 16 data attributes, and 14 object attributes, with a total of 125,591 nodes and 547,224 semantic relationships. Specific details regarding the triplet semantic relationships are provided in **Table 1**.

**Table 1:** Statistics of triples in knowledge graph for traits regulating-genes.

head entity; relation; tail entity	head entity	triples	tail entity	data source
(protein; associates with; trait)	1060	1235	6	PubMed
(protein; homologous to; protein)	1060	191221	75863	UniProt
(protein; interacts with; protein)	351	1150	351	STRING
(protein; corresponding to; gene)	76427	76427	28761	Phytozome; RAPdb
(protein; identify with; gene symbol)	18761	18761	12613	UniProt
(protein; involves in; signal pathway)	3345	3486	235	UniProt
(protein; located in; subcellular localization)	24317	41099	1885	UniProt
(protein; has protein domain; domain)	2211	2424	322	UniProt
(protein; belongs to; protein family)	41422	57582	642	Pfam
(gene; located in; cellular component)	20673	32897	389	Ensembl plants; GO
(gene; performs; molecular function)	25990	63750	1176	Ensembl plants; GO
(gene; involves in; biological process)	21794	46905	2105	Ensembl plants; GO
(gene; involves in; metabolic pathway)	4765	4777	604	Ensembl plants; KEGG
(gene; encodes the enzyme type; enzyme)	5094	5510	423	Ensembl plants; KEGG

UniProt, Universal Protein Resource; KEGG, Kyoto Encyclopedia of Genes and Genomes; Ensemble, European Molecular Biology Laboratory’s European Bioinformatics Institute; RAPdb, Rice Genome Annotation Project; GO, Gene Ontology.

### 3.2 Prediction of genes regulating-traits

**Figure 2:**
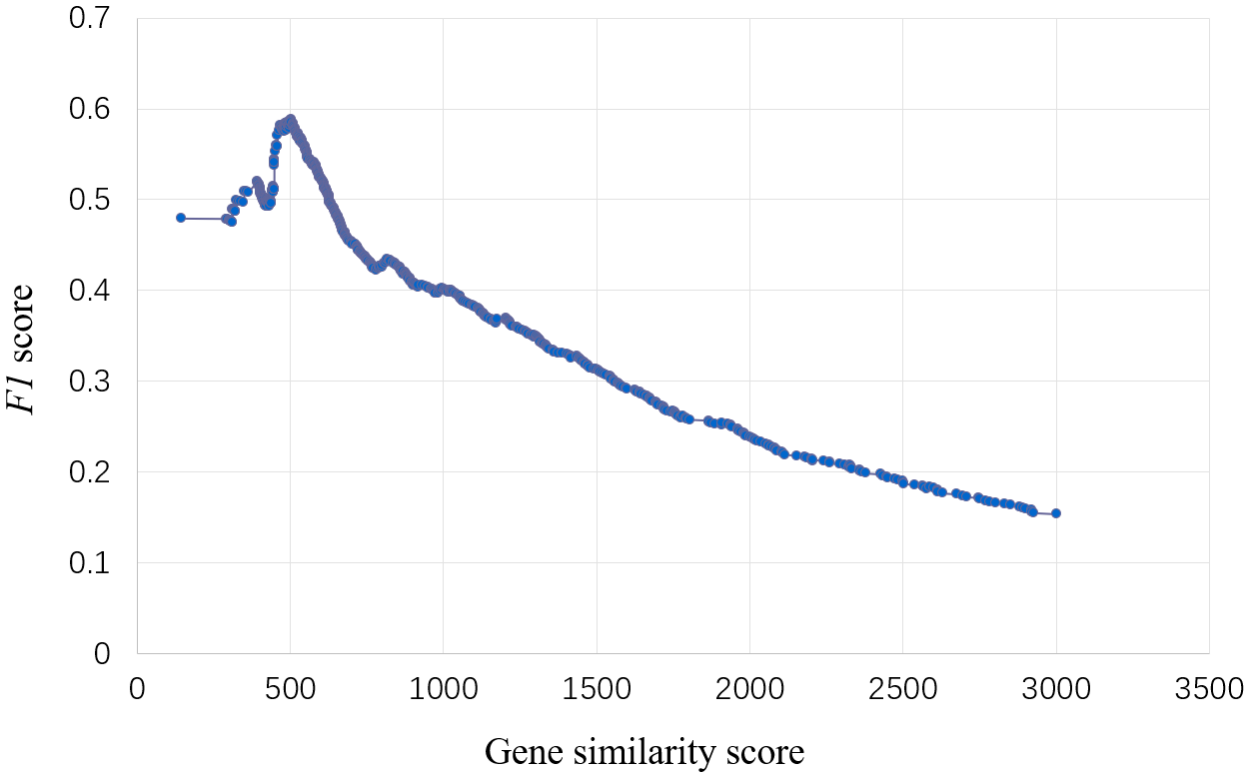
Select threshold of the model for traits regulating-genes. The abscissa is the gene similarity score, the ordinate is the F1 value, and the model has the best effect when the F1 value reaches the peak, and the corresponding abscissa similarity score is the threshold.

To predict the genes regulating specific traits, we obtained 885 gene-trait association datasets published from 1988 to 2023 and divided them into training set and validation set. In the training set, F1 value used as the threshold filtering index. When F1 peaked, the corresponding gene node similarity score was 502.36 (**Figure 2**).

**Figure 3:**
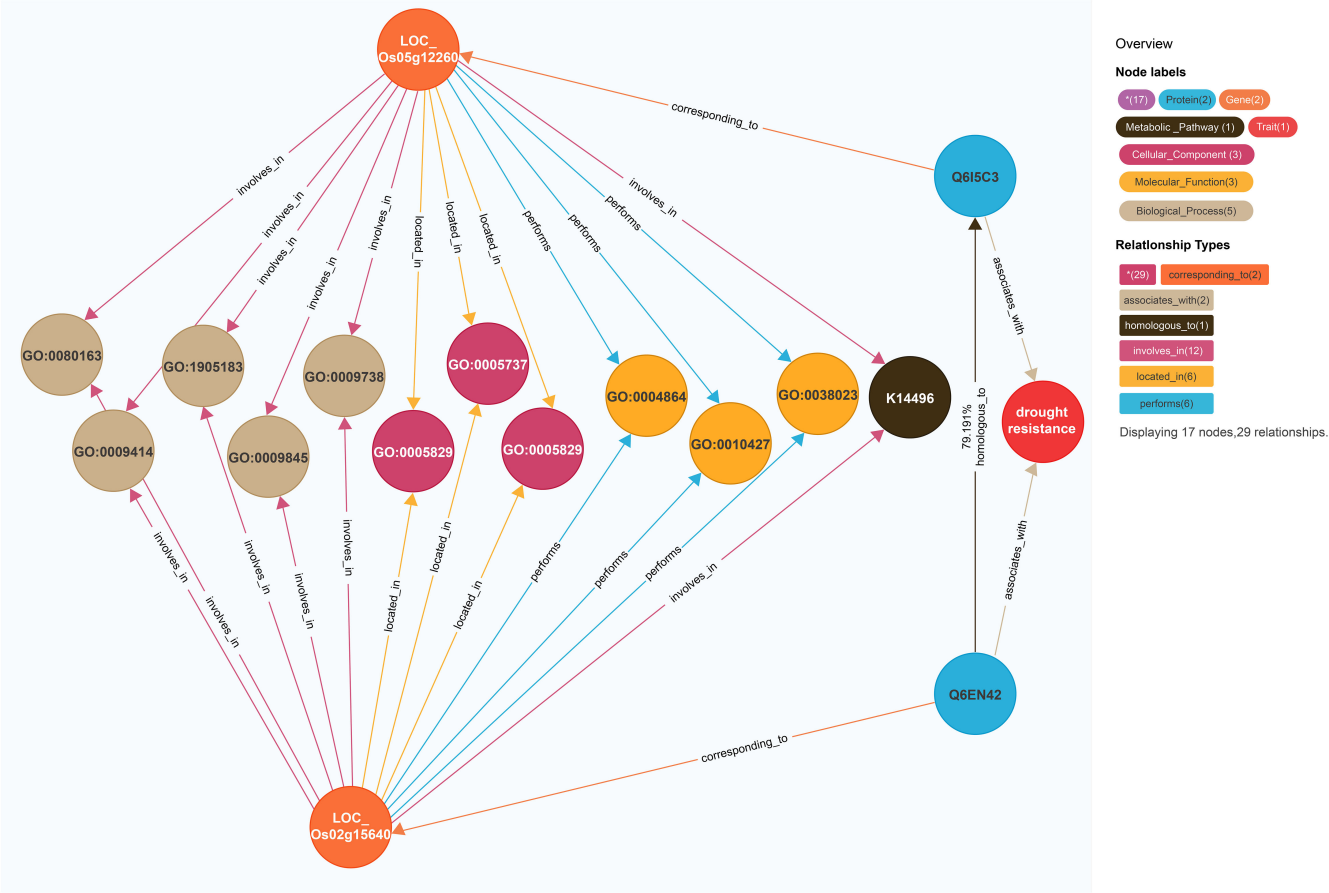
Prediction of *LOC_Os02g15640*’s regulatory traits. The different-colored circles and arrow lines in the legend in the upper right corner represent different entity types and relationship types, respectively, and the numbers in parentheses indicate the number of nodes and relationships of different types. For example, the red circle represents the “trait” node, and the number is 1; The light blue circle represents the “protein” node, which has a quantity of 2; The orange circle represents the “gene” node, which has several 2. Taking the relationship type as an example, the brown arrow line represents the “homologous to” relationship type, and the number is 1; The light blue arrow line represents the exercise “(performs)” relationship type, with a quantity of 4; The number of co-connected coincident nodes between the gene entity *LOC_Os05g12260* and the gene entity *LOC_Os02g15640* was 12, the number of coincident node classes was 4, and the corresponding proteins were Q6I5C3 and Q6EN42, and the similarity between them was 79.191.

On the basis of the model for predicting traits regulating-genes proposed in this study, the rice gene *LOC_Os05g12260* (**Figure 3**) was revealed to be associated with drought resistance traits in 2014 (Kim et al., 2014). This model was also used to calculate the similarity between the gene entities *LOC_Os05g12260* and *LOC_Os02g15640*. There were 13 coincident nodes between the two gene entities. Additionally, there were four coincident node categories. The similarity score for the proteins (Q6I5C3 and Q6EN42, respectively) encoded by *LOC_Os05g12260* and *LOC_Os02g15640* was 79.191. The similarity score for the two gene entities (3,801.17; i.e., 12 × 4 × 79.191) exceeded the gene similarity threshold (502.36). We speculated that *LOC_Os02g15640* is likely associated with drought resistance. A previous study indicated that the overexpression of this gene significantly improves the drought and cold tolerance of rice (Tian et al., 2015). Accordingly, the findings of the current study reflect the utility of the model for predicting traits regulating-genes.

### 3.3 Mining the elite polyphenotype genes

To identify elite polyphenotype genes, the knowledge mining method for predicting links was used to establish the implicit associations between gene entities and multiple trait entities on the basis of the model for predicting traits regulating-genes. A total of 4,447 polyphenotype genes for 31 trait combinations were mined (**Table 2**), including 1,925 functional known genes (**Supplementary Table 1**) and 2,522 functional unknown genes (**Supplementary Table 2**).

**Table 2:** The number of polyphenotype genes related to different combination traits.

Traits	Numbers
salt_resistance, drought_resistance	962
disease_resistance, salt_resistance	800
disease_resistance, drought_resistance	378
disease_resistance, plant_height_reduce	168
salt_resistance, plant_height_reduce	156
disease_resistance, insect_resistance	142
disease_resistance, grain_weight_increase	108
salt_resistance, insect_resistance	98
grain_weight_increase, plant_height_reduce	43
plant_height_reduce, insect_resistance	6
grain_weight_increase, insect_resistance	2
insect_resistance, drought_resistance	2
plant_height_reduce, drought_resistance	1
grain_weight_increase, salt_resistance	12
disease_resistance, salt_resistance, drought_resistance	1173
disease_resistance, salt_resistance, plant_height_reduce	78
disease_resistance, grain_weight_increase, plant_height_reduce	74
salt_resistance, insect_resistance, drought_resistance	39
disease_resistance, salt_resistance, insect_resistance	27
grain_weight_increase, salt_resistance, plant_height_reduce	19
disease_resistance, plant_height_reduce, insect_resistance	8
disease_resistance, grain_weight_increase, insect_resistance	3
grain_weight_increase, salt_resistance, drought_resistance	2
disease_resistance, grain_weight_increase, drought_resistance	1
disease_resistance, insect_resistance, drought_resistance	1
disease_resistance, grain_weight_increase, salt_resistance, drought_resistance	52
disease_resistance, salt_resistance, insect_resistance, drought_resistance	41
disease_resistance, salt_resistance, plant_height_reduce, drought_resistance	11
disease_resistance, grain_weight_increase, salt_resistance, plant_height_reduce	6
disease_resistance, grain_weight_increase, plant_height_reduce, insect_resistance	1
grain_weight_increase, disease_resistance, drought_resistance, plant_height_reduce, salt_resistance	34

**Figure 4:**
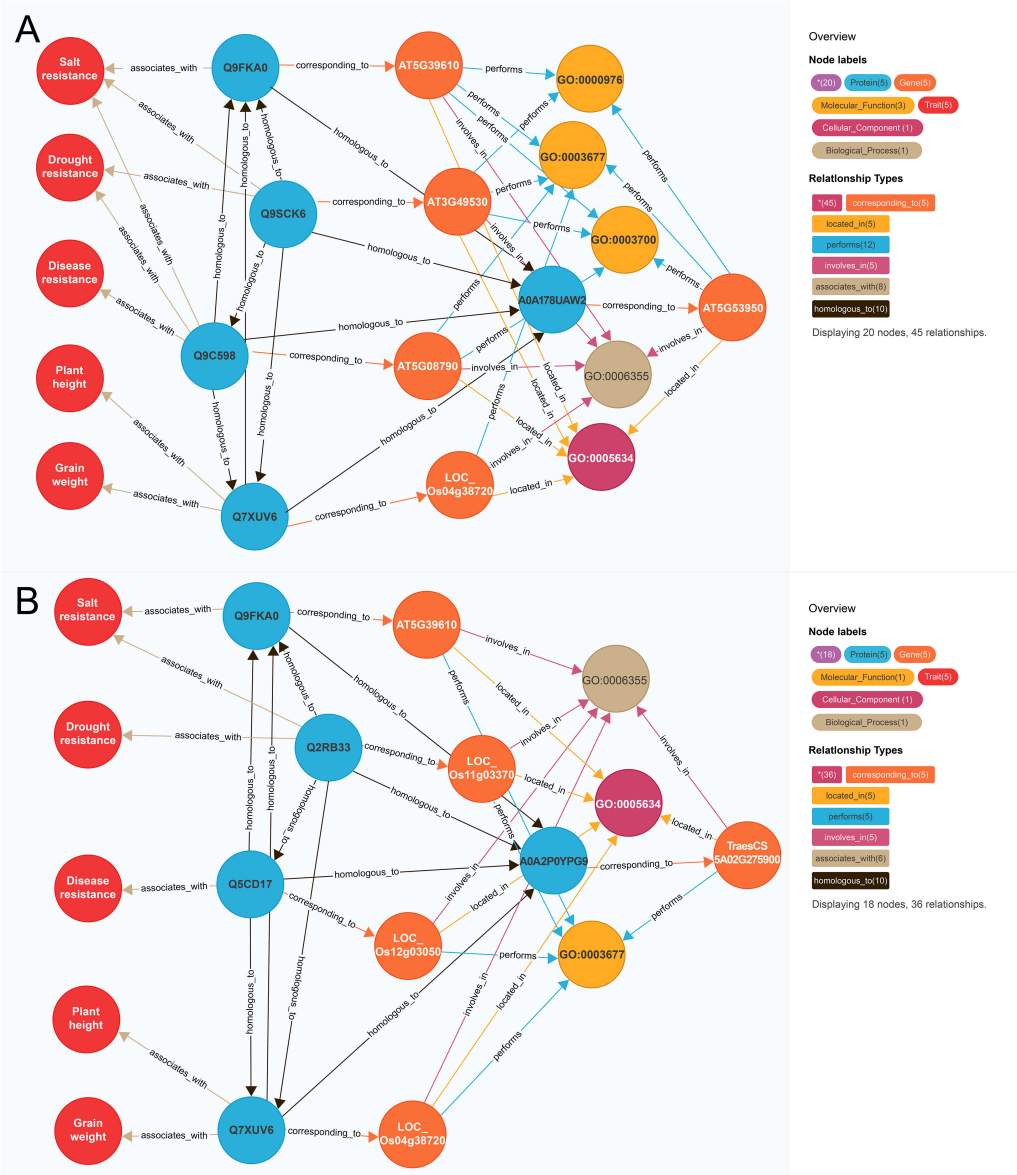
Mining polyphenotype gene. **(A)** Mining of functional known genes *AT5G53950*; **(B)** Mining of functional unknown genes *TraesCS5A02G275900*. The differently colored circles and arrow lines in the legend represent different entity types and relationship types respectively, the numbers in parentheses indicate the number of nodes and relationships of different types. Taking **(A)** as an example, the red circle represents the “trait” node, and the number is 5. The light blue circle represents the “protein” node, which is in number 5. The orange circle represents the “gene” node, which has several 5. The brown arrow line represents the “homologous to” relationship type, and the number is 10.

To further illustrate this model, we selected functional known genes and functional unknown genes as examples respectively. For instance, we identified the functional known gene *CUC2* (*AT5G53950*) which is an elite polyphenotype gene (**Figure 4A**). In *A. thaliana*, *AT5G39610* is associated with salt resistance (Zhao et al., 2022), *AT3G49530* is associated with drought resistance (Kim et al., 2012), and *AT5G08790* is associated with disease resistance (Viswanath et al., 2023). In rice, *LOC_OS04G38720* influences plant height and grain weight (Chen et al., 2015; Jiang et al., 2018). The similarity scores between *AT5G53950* and the above-mentioned four genes (1,156.86, 671.88, 653.84, and 645.00, respectively) were higher than the threshold (502.36), implying *AT5G53950* may regulate salt resistance, drought resistance, disease resistance, plant height, and grain weight. Recent studies confirmed that the *A. thaliana* gene *AT5G53950* contributes to drought resistance (Meng et al., 2023), salt resistance (Li et al., 2023), grain weight (Liu et al., 2023a), and disease resistance (Su et al., 2023), providing further evidence of the utility of our model for predicting traits regulating-genes.

Among the functional unknown genes, *TraesCS5A02G275900* was selected as a representative example (**Figure 4B**). The *A. thaliana* gene *AT5G39610* is associated with salt resistance (Zhao et al., 2022). In rice, *LOC_Os11g03370* is associated with drought resistance and salt resistance (Zheng et al., 2009), whereas *LOC_Os12g03050* is associated with disease resistance (Chromosomes and Consortia, 2005) and *LOC_Os04g38720* is correlated with plant height and grain weight (Chen et al., 2015; Jiang et al., 2018). The similarity scores between the wheat gene *TraesCS5A02G275900* and the above-mentioned four genes were 689.52, 602.25, 580.00, and 675.00, respectively, which exceeded the threshold (502.36). Hence, *TraesCS5A02G275900* may regulate salt resistance, drought resistance, disease resistance, plant height, and grain weight, but the possibility *TraesCS5A02G275900* is an elite polyphenotype gene remains to be experimentally verified.

### 3.4 Identification of ideal polyphenotype genes for target traits

In response to the demand for mining suitable polyphenotype genes affecting target traits, we comprehensively ranked the elite polyphenotype genes according to the similarities between unknown and known genes. Specifically, the similarity scores of the unknown genes for each regulated trait were ranked, after which the polyphenotype genes were comprehensively ranked in terms of the target traits. For example, during the cultivation of new high-quality crop varieties, dwarfism-related traits suitable for mechanized harvesting as well as high yields and disease resistance are ideal. Thus, we selected the top 10 genes (10 functional known genes and two functional unknown genes) for the regulated traits (**Table 3**).

**Table 3:** Top10 polyphenotype genes related to plant height, grain weight, and disease resistance.

Gene_ID	R_PH	R_GW	R_DiR	R_M	R_C	V_T
*LOC_Os08g39890*	1	1	1	1.0	1	known
*Zm00001d031451*	8	5	4	5.7	2	known
*Zm00001d052890*	10	6	5	7.0	3	known
*Zm00001d017742*	1	2	24	9.0	4	known
*LOC_Os02g47280*	2	1	29	10.7	5	known
*AT2G22840*	15	15	6	12.0	6	known
*Zm00001d018260*	7	4	30	13.7	7	unknown
*TraesCS6D02G245300*	16	11	27	18.0	8	unknown
*LOC_Os04g51190*	24	13	18	18.3	9	known
*AT1G27370*	21	18	16	18.3	9	known
*AT2G36400*	11	7	38	18.7	10	known
*AT4G37740*	31	8	17	18.7	10	known

R_PH, rank in plant height; R_GW, rank in grain weight; R_DiR, rank in disease resistance; R_M, mean rank in plant height, grain weight, and disease resistance; R_C, comprehensive rank according to R_M; V_T, verified trait, The term “known” indicates that this gene has been verified with at least one of the three traits in the literature, while the term “unknown” indicates that this gene has not been verified in the literature.

Among the selected genes, the functional known gene *IPA1* (*LOC_Os08g39890*) (**Table 3**) may be an ideal polyphenotype gene that likely regulates plant height, grain weight, and disease resistance. This gene reportedly encodes a plant-specific SBP-box domain-containing transcription factor that functions as a major regulator of the rice plant type (Jiao et al., 2010). The *ipa1-1D* and *ipa1-2D* alleles can increase the number of panicles, decrease the number of ineffective tillers, thicken stems, enhance root system development, and ultimately significantly increase the yield; thus, they have been widely used for breeding elite rice varieties (Wang et al., 2018). In addition, *IPA1* also plays an important regulatory role influencing disease resistance and environmental adaptations (Liu et al., 2019; Chen et al., 2023). Hence, our prediction results were supported by the published literature, which further illustrates the reliability of our model for predicting traits regulating-genes. We also predicted that the functional unknown genes *Zm00001d018260* and *TraesCS6D02G245300* are associated with plant height, grain weight, and disease resistance.

We also mined for the polyphenotype genes that simultaneously regulate salt resistance, plant height, grain weight, drought resistance, and disease resistance. The top 10 genes associated with these traits consisted of seven functional known genes and four functional unknown genes. Of the genes predicted to affect salt resistance, plant height, grain weight, drought resistance, and disease resistance, *AT5G53950* was ranked first (**Table 4**). Recent research revealed that this gene is important for regulating leaf development and salt resistance (Li et al., 2023), while also contributing to the response to drought stress via the ubiquitin pathway (Meng et al., 2023). Another recent study confirmed that this gene modulates fruit yield and quality by regulating shoot apical meristem inflorescence development and fruit ripening (Liu et al., 2023a). Moreover, this gene belongs to the *EIL* family, which can effectively protect against disease-related plant decay and is widely used to enhance crop disease resistance (Su et al., 2023). Hence, according to published research, this gene is indeed related to salt resistance, drought resistance, disease resistance, and grain weight. In addition, this gene may also regulate plant height, which is a potential subject discovery.

**Table 4:** Top10 polyphenotype genes related to salt resistance, plant height, grain weight, drought resistance, and disease resistance.

Gene_ID	R_SR	R_PH	R_GW	R_DR	R_DiR	R_M	R_C	V_T
*AT5G53950*	4	7	5	6	8	6.0	1	known
*AT3G29035*	1	13	8	4	4	6.0	2	known
*TraesCS5A02G275900*	10	4	4	9	10	7.4	2	unknown
*TraesCS2D02G324700*	11	1	2	18	12	8.8	3	known
*LOC_Os04g38720*	12	3	10	10	11	9.2	4	known
*AT3G15510*	7	16	14	5	5	9.4	5	known
*AT1G01720*	3	15	26	2	2	9.6	6	known
*TraesCS2A02G338300*	13	1	3	19	15	10.2	7	unknown
*AT1G77450*	2	30	19	1	1	10.6	8	known
*TraesCS4D02G071200*	14	9	7	12	13	11.0	9	unknown
*TraesCS4A02G242700*	15	8	6	14	16	11.8	10	unknown

R_SR, rank in salt resistance, R_PH, rank in plant height; R_GW, rank in grain weight; R_DR, rank in drought resistance; R_DiR, rank in disease resistance; R_M, mean rank in plant height, grain weight, and disease resistance; R_C, comprehensive rank according to R_M; V_T, verified trait, The term “known” indicates that this gene has been verified with at least one of the five traits in the literature, while the term “unknown” indicates that this gene has not been verified in the literature.

### 3.5 Verification of the polyphenotype gene mining results

**Figure 5:**
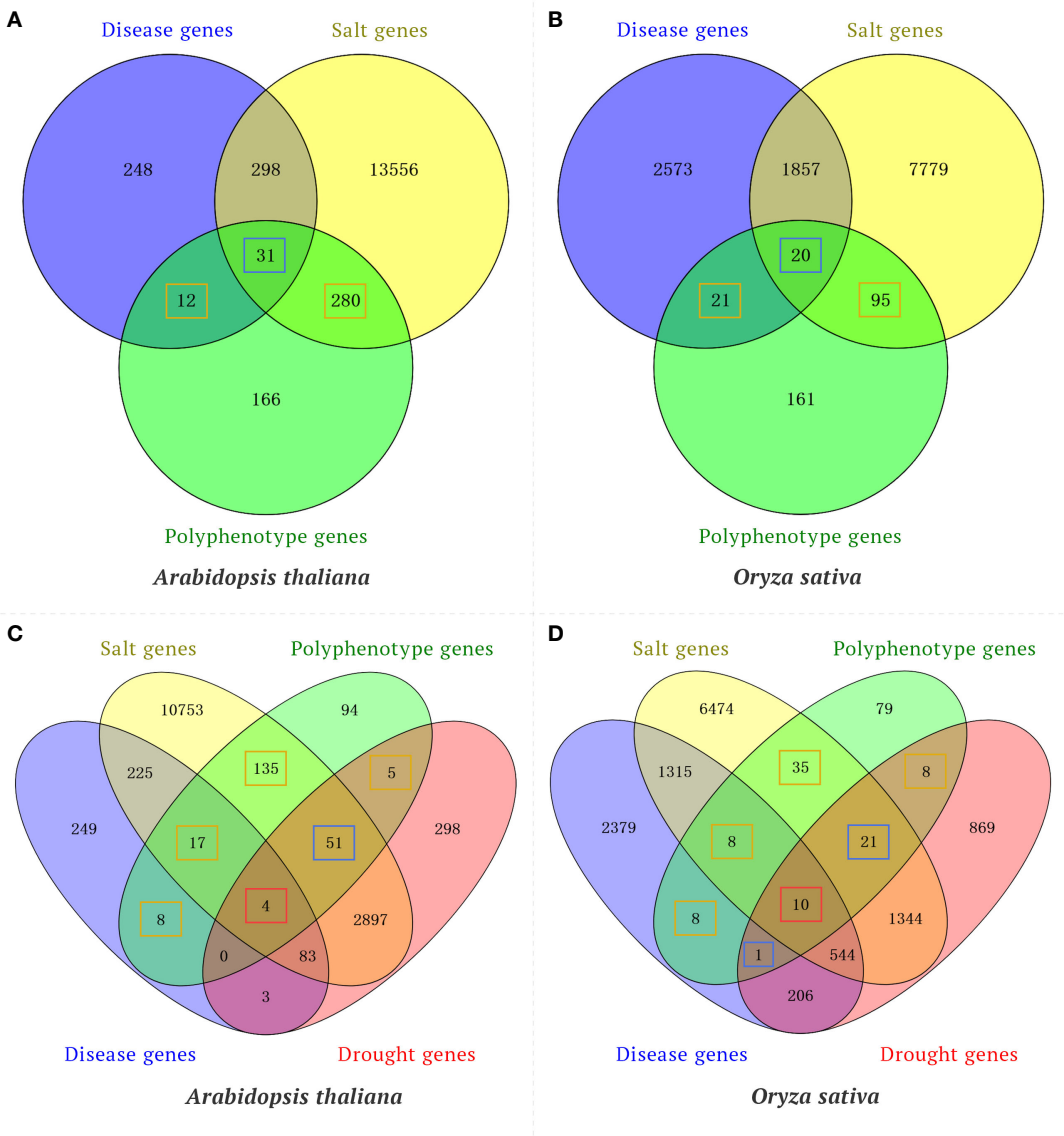
Venn diagram analysis of polyphenotype genes and differentially expressed genes (DEGs) in response to different phenotypes. **(A)** Venn diagram analysis of the polyphenotype genes and transcriptome genes related to disease and salt in *Arabidopsis*. **(B)** Venn diagram analysis of polyphenotype genes and transcriptome genes related to disease and salt in rice. **(C)** Venn diagram analysis of polyphenotype genes and transcriptome genes related to disease, salt and drought in *Arabidopsis*. **(D)** Venn diagram analysis of polyphenotype genes and transcriptome genes related to disease, salt, and drought in rice. The orange boxes represent polyphenotype genes associated with a single phenotype in transcriptome datasets, the blue boxes represent polyphenotype genes associated with two phenotypes in transcriptome datasets, and the red boxes represent polyphenotype genes associated with three phenotypes in transcriptome datasets.

To confirm the utility of the knowledge graph-based model for predicting traits regulating-genes, a time-slicing method was used to examine the published literature to screen the validation set for gene pairs with a similarity score greater than or equal to the threshold (502.36). Several indices (accuracy, precision, recall, and F1 value) were used to evaluate the prediction results. For the test set, the model accuracy was 0.89, the precision rate was 0.91, the recall rate was 0.96, and the F1 value was 0.94. These results reflect the utility of the knowledge graph-based model for predicting traits regulating-genes. Moreover, the rice polyphenotype gene *IPA1* (*LOC_Os08g39890*) and the *A. thaliana* polyphenotype gene *CUC2* (*AT5G53950*) were also verified by published research findings. However, the wheat gene *TraesCS5A02G275900* is a potential polyphenotype gene that has not been reported. These results are indicative of the reliability of the method used for identifying elite polyphenotype genes. In addition, to verify the reliability of polyphenotype genes predicted in our model, we performed Venn diagram analysis between the polyphenotype gene datasets (consists of genes that are predicted to have multiple phenotypes by our model) and the transcriptome gene datasets (consists of genes that were differential expression in response to disease, drought or salt). For example, by using our model, we identified 489 and 297 polyphenotype genes associated with both disease and salt tolerance in *Arabidopsis* and rice, respectively. Venn diagram analysis suggested that 292 and 116 polyphenotype genes were associated with a single phenotype (disease or salt) in transcriptome datasets of *Arabidopsis* and rice, respectively (**Figures 5A, B**). In addition, 31 and 20 polyphenotype genes were associated with both disease and salt in transcriptome datasets of *Arabidopsis* and rice, respectively (**Figures 5A, B**). Our results suggested that approximately 66% and 46% of polyphenotype genes were verified by transcriptome data in *Arabidopsis* and rice, respectively. Furthermore, our model predicted 314 and 170 polyphenotype genes associated with disease, salt and drought in *Arabidopsis* and rice, respectively. We found that approximately 70% and 54% polyphenotype genes were identified in the transcriptome datasets of *Arabidopsis* and rice, respectively (**Figures 5C, D**). Taking together, most of the polyphenotype genes were verified in transcriptome analysis, our model provides a new method for researchers to narrow down candidate genes.

## 4 Discussion

In this study, we constructed a knowledge graph for traits regulating-genes, this knowledge graph described the knowledge organization system for multi-dimensional scientific data association of cross-species genes and traits. In addition, based on the knowledge graph, we generate a new model for identification of elite polyphenotype genes. Furthermore, we tested this model by using gene-trait association datasets from 4 species, including Arabidopsis, wheat, rice, and maize. Our results suggested that the model is applicable for detecting ideal functional known and functional unknown polyphenotype genes. The existing methods for genome-wide association analysis (Garcia et al., 2019), quantitative trait locus mapping (Jiang et al., 2023), and mixed pool group analyses (Liu et al., 2023b) establish the association between genomic data and phenotypic data. These methods only can localize candidate genes in one segment and clarify the associations between genes and individual traits, but they are less than ideal for identifying elite polyphenotype genes. Hence, our study proposed a new method to identify elite polyphenotype genes for the development of new crop varieties with enhanced yield and superior quality.

In fact, knowledge graphs have been applied in crop sciences in previous studies. The Rothamsted Research institute constructed KnetMiner, which is a gene network discovery platform for agricultural researchers to explore and clarify the complex crop traits in different plant species (Hassani-Pak et al., 2021). In addition, the CIRAD (French Agricultural Research Centre for International Development) use the Semantic Web technology to develop a knowledge graph named AgroLD, which integrates scientific data on different plant species to provide new scientific hypotheses about functional genes (Larmande et al., 2022). However, both of them only analyze the correlation between genes and individual traits, so it is difficult to realize the mining of polyphenotype genes. In our study, we constructed a knowledge graph for traits regulating-genes comprising 13 entities, 16 entity data attributes, and 14 entity object attributes, with a total of 125,591 nodes and 547,224 semantic relationships across 4 species (Arabidopsis, wheat, rice, and maize). The key in our knowledge graph is the establishment of homologous protein object attributes, the association and fusion of scientific data in cross-species is realized. In addition, our knowledge graph provides data support to illustrate the relationships among the multi-dimensional scientific data in cross-species genes and traits (**Figure 4**). Our knowledge graph for traits regulating-genes can provide hypotheses about the association between genes and multiple traits based on multi-dimensional scientific data. Our study drives the mining of elite polyphenotype genes from hypothesis-driven passive exploration to data-driven active knowledge discovery.

Meanwhile, there are some methods about gene mining. For example, whole genome association analyses to locate gene loci related to plant height (Guo et al., 2022), yield (Li et al., 2021), and disease resistance (Tsai et al., 2020). They only localize the relevant gene loci into a segment containing many candidate genes. In our study, we developed a model for predicting traits regulating-genes. This model can give the probability score of trait regulating-genes on the basis of a multi-dimensional scientific data analysis (**Figure 3**). For example, by combining elite traits (e.g., plant height, grain weight, and disease resistance) as search terms, a comprehensive score of the probability of the genes regulating specific traits can be calculated using multi-dimensional scientific data for cross-species genes and traits, with the top candidate polyphenotype genes identified according to the scores. The key in our model is that combines the entity semantic association characteristics and graph structure characteristics about multi-dimensional scientific data between genes and traits. That is to say, in order to achieve the recommendation of polyphenotype genes, our model integrates the data attribute information of gene nodes and the topological relationship information of gene nodes. Consequently, our model can provide an evidence-based approach with specific probability score to rank candidate genes for target traits, which can to narrow down the candidate genes associated with multiple phenotypes. Compared with transcriptome or other traditional methods for gene mining, our model provided more possibilities and effectiveness in mining polyphenotype genes.

To sum up, our study provides an important model for the mining and recommendation of elite polyphenotype genes for target traits in crops. Of course, researchers need perform further molecular biology experiments and transgenic experiments to verify the mining results of polyphenotype genes. In addition, the underlying principle of our model to mine polyphenotype genes based on the knowledge graph for integrating scientific data related to genes across species. With the deepening of research on plant functional genes, we also need to increase the gene-related scientific data volume and update newly published data, to make our model more broadly applicable.

## 5 Conclusion

In this study, a model for predicting traits regulating-genes was constructed using a knowledge graph for traits regulating-genes. The accuracy of the model for predicting traits regulating-genes was 0.89, the precision rate was 0.91, the recall rate was 0.96, and the F1 value was 0.94, implying the traits regulating-genes were relatively accurately predicted on the basis of a multi-dimensional scientific data analysis. The comprehensive ranking of polyphenotype genes based on the model mined elite polyphenotype genes for different trait combinations. This study provides important data for future investigations of the molecular mechanisms regulating crop traits, with implications for crop breeding and research. Furthermore, our model represents a new tool for identifying elite polyphenotype genes.

## Data availability statement

The original contributions presented in the study are included in the article/**Supplementary Material**. Further inquiries can be directed to the corresponding author.

## Author contributions

DZ: Data curation, Formal analysis, Investigation, Methodology, Resources, Writing – original draft, Writing – review & editing. RZ: Funding acquisition, Project administration, Supervision, Writing – review & editing. GX: Supervision, Writing – review & editing. YK: Project administration, Supervision, Writing – review & editing. WM: Data curation, Writing – review & editing.
